# A single‐center, open‐label, randomized cross‐over study to evaluate the pharmacokinetics and bioavailability of once‐daily prolonged‐release formulations of tacrolimus in de novo liver transplant recipients

**DOI:** 10.1002/iid3.537

**Published:** 2021-09-24

**Authors:** Uta Herden, Martina Sterneck, Bettina M. Buchholz, Eike G. Achilles, Armin Ott, Lutz Fischer

**Affiliations:** ^1^ Department of Visceral Transplantation University Medical Center Hamburg‐Eppendorf Hamburg Germany; ^2^ Department of Medicine University Medical Center Hamburg‐Eppendorf Hamburg Germany; ^3^ Institute of Medical Informatics, Statistics and Epidemiology Technische Universität München Munich Germany

**Keywords:** liver transplantation, pharmacokinetics, prolonged‐release formulations, tacrolimus

## Abstract

**Background:**

The narrow therapeutic window of tacrolimus (Tac) requires intense drug monitoring to achieve adequate efficacy while minimizing dose‐related toxicities. Once‐daily formulations of Tac (LCP‐Tac and PR‐Tac) have been recently designed for higher bioavailability and a more consistent exposure over time, as opposed to the twice‐daily, administered immediate‐release formulation of Tac (IR‐Tac).

**Methods:**

This single‐center, open‐label, randomized cross‐over pharmacokinetic (PK) study compares extended‐release LCP‐Tac with the prolonged‐release formulation of tacrolimus (PR‐Tac) in adult de novo liver transplant recipients. Eligible patients were screened and randomized 1:1 to the two treatment arms up to 30 days after liver transplantation. Patients were administered either LCP‐Tac or PR‐Tac for 14 days followed by another 14‐day time interval of the other once‐daily Tac medication. A 24hr‐PK profile was obtained at the end of each time interval.

**Results:**

Nine patients (45%) completed the study resulting in a total of 18 Tac PK profiles. Overall, the profile of the mean concentrations indicated a flattened kinetic of LCP‐Tac compared to PR‐Tac, especially in the first 3 h after drug intake. The average cumulative dose per day to achieve equivalent trough levels was approximately 25% lower for LCP‐Tac (8.7 mg) than for PR‐Tac (11.7 mg). LCP‐Tac resulted in a longer t_max_ and fewer peak‐to‐trough fluctuations compared to PR‐Tac.

**Conclusion:**

Despite methodological weaknesses that limit the conclusions, we have found a more consistent drug exposure for LCP‐Tac in de novo LT recipients. LCP‐Tac demonstrated a greater bioavailability compared to PR‐Tac.

## Graphical abstract

This is a single‐center, open‐label, randomized cross‐over pharmacokinetic (PK) study, designed to compare LCP‐Tac with the established once‐daily prolonged‐release formulation of tacrolimus (PR‐Tac) in adult de novo liver transplant recipients. As a result, we have more consistent drug exposure for Envarsus® and, in particular, a lower medication requirement to achieve equivalent trough levels.

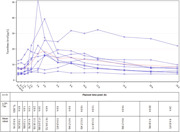

## 1 INTRODUCTION

The calcineurin inhibitor (CNI) tacrolimus (Tac) remains the cornerstone of immunosuppressive therapy after solid organ transplantation. Approximately 90% of adult kidney and liver transplant recipients receive Tac as part of their immunosuppressive regimen.^1^, ^2^ Although Tac potently prevents graft rejection, significant between‐subject and within‐subject variability in absorption and between‐subject variability in metabolism present significant challenges in the utilization of CNIs in clinical practice.^3^ Consequently, transplant recipients require intense therapeutic drug monitoring to maintain therapeutic target trough levels and minimize toxicity, especially during the first months after transplantation.

The narrow therapeutic window of Tac may otherwise easily lead to undesirable effects such as over‐ or under‐immunosuppression, risking either drug‐related toxicity or acute rejection episodes early after liver transplantation.^4^, ^5^ Usually administered as a standard twice‐daily formulation every 12 h, the immediate‐release formulation of Tac (IR‐Tac) exhibits a relatively low and variable bioavailability.^6^ A potential strategy in patients who have demonstrated variability in immunosuppressive exposure under twice‐daily IR‐Tac (e.g., Prograf®, Tacrolimus HEXAL®, Crilomus®, Tacpan®) is to switch to a prolonged‐release formulation of Tac (PR‐Tac). One advantage of prolonged‐release formulations is the simplification of the therapy regimen by once‐daily dosing, which can be beneficial to the medication adherence of the patient.^7^

Advagraf® was the first PR‐Tac formulation introduced into routine clinical practice. To achieve a sustained release of Tac, galenics were changed by excipients such as ethylcellulose.^6^ The other once‐daily Tac formulation is using the MeltDose drug delivery technology (LCP‐Tac, Envarsus®) and has been authorized by the European Medicines Agency (EMA) in 2014. LCP‐Tac is approved for the prevention of transplant rejection in adult kidney or liver allograft recipients and for the treatment of allograft rejection in adult patients who fail to respond to other immunosuppressants. The MeltDose technology allows reducing the particle size of Tac to the smallest possible unit (<0.1 µm diameter), which results in enhanced bioavailability by better dissolution and absorption.^8^, ^9^ As shown in an open, multicentric, and prospective study with 57 stable liver transplant recipients, the greater bioavailability of LCP‐Tac allowed not only once‐daily dosing but achieved also comparable systemic exposure and Tac trough levels at a dose approximately 30% less than the total daily dose of IR‐Tac.

The objective of our study is to analyze the differences in the pharmacokinetic (PK) profile of LCP‐Tac and PR‐Tac in de novo liver transplant recipients in a prospective randomized trial. Investigating the dose of the two formulations required to achieve therapeutic trough levels in de novo administration may help to gain additional information on dose titration strategies in daily clinical practice.

## 2 PATIENTS AND METHODS

The primary objective of this study was to determine the equivalent dose of LCP‐Tac to yield the same target trough level as achieved with PR‐Tac and to assess the conversion ratios of Tac in adult de novo liver transplant recipients switching between LCP‐Tac and PR‐Tac. Secondary endpoints included the comparative evaluation of the LCP‐Tac‐ and PR‐Tac‐associated PK profiles and bioavailability, and the assessment of the safety profiles of the two Tac formulations.

### 2.1 Study design

This was an investigator‐initiated, single‐center, open‐label, randomized, and controlled clinical cross‐over Phase III study (EudraCT Number: 2015‐002935‐16) conducted at the University Medical Center Hamburg‐Eppendorf, Department of Visceral Transplantation, Germany.

**Figure 1:**
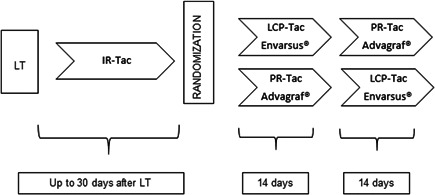
Cross‐over study design. IR‐Tac, immediate‐release tacrolimus; LCP‐Tac, MeltDose tacrolimus (Envarsus®, 0.75, 1, and 4 mg tablets); LT, liver transplantation; PR‐Tac, prolonged‐release tacrolimus (Advagraf®, 0.5, 1, 3, and 5 mg capsules)

Eligible patients were screened and randomized 1:1 to the two treatment arms after a run‐in period with standard IR‐Tac twice daily via nasogastric tube (Modigraf®) or orally (Prograf®) for up to 30 days after liver transplantation. Patients were then switched to either LCP‐Tac (Envarsus®) or PR‐Tac (Advagraf®) for 14 days followed by another 14‐day time interval of the other once‐daily Tac medication (cross‐over concept) (Figure 1). Tac trough target levels were individually defined for each patient usually ranging between 6 and 10 ng/ml. Trough level adjustment was based on liver function tests, risk of rejection, comorbidities such as renal failure or infections, time elapsed since transplantation, and clinical experience.

All patients received induction therapy with basiliximab 20 mg on postoperative Days 0 and 4. Steroids were given according to center practice with a fast taper to 5–10 mg of Prednisolon orally after 1 week. No further immunosuppression was permitted during the study period.

### 2.2 Compliance with ethical guidelines

The study was approved by the Ethics Committee of the University Medical Center Hamburg‐Eppendorf, Germany. All patients gave written consent for participation in the study.

### 2.3 Inclusion and exclusion criteria

Female or male recipients of a liver graft from a deceased or living donor within the last 30 days were eligible if they were aged ≥18 years and received a twice‐daily Tac‐based immunosuppressive treatment at the time of randomization. Patients receiving combined organ transplants or ABO‐incompatible allografts and patients with impaired kidney function requiring dialysis were excluded. Furthermore, patients with known hypersensitivity to any of the drugs used in the study, inability to take oral medication at the time of randomization, or comedication with drugs known to strongly interact with the CYP3A4 system were disqualified to participate in this study. In all cases, the eligibility of patients was assessed within the data review meeting (DRM) (blind DRM).

### 2.4 Sample collection and bioanalytical methods

During the 2‐week intervals, study visits were performed every 2–4 days to analyze Tac through blood levels (C_0_) and to adjust the study medication if necessary. A conversion ratio of either 0.7 (LCP‐Tac) or 1.0 (PR‐Tac) was used to determine the first dose of each formulation when switching from IR‐Tac or between the two once‐daily Tac formulations.^10^, ^11^ To evaluate the PK profiles of LCP‐Tac in comparison to PR‐Tac, a 24‐h PK assessment was conducted on Day 14 of receiving each formulation. Blood samples (2.7 ml) were taken as trough level (predose, within 30 min before administration of the medication) and at 0.5, 1.0, 1.5, 2.0, 3.0, 4.0, 6.0, 8.0, 10.0, 12.0, 16.0, 20.0, and 24.0 h after oral intake of the immunosuppressant. The 24‐h PK sample collection required a steady state of Tac trough levels (no dose adjustment >25% performed at the next‐to‐last study visit, and no dose adjustment at all at the last study visit; otherwise PK profiling was postponed until a steady state was reached as assessed by additional study visits). Study visits took place in the outpatient clinic for patients already discharged after liver transplantation, while all study participants were readmitted to the hospital for assessment of PK profiles. Tac whole blood concentrations were analyzed by tandem mass spectrometry (liquid chromatography‐mass spectrometry/mass spectrometry [LC‐MS/MS]) at the Clinical Chemistry Laboratories of the University Medical Center Hamburg‐Eppendorf, Germany.

### 2.5 PK population

As set out in the study protocol, completion of both 14‐day intervals with LCP‐Tac and PR‐Tac was required for inclusion in the PK population. Patients were excluded from PK analysis if the plasma concentration‐time profile might have been unreliable due to gastrointestinal disturbances such as vomiting and diarrhea or if concomitant medications were used that could interact with the absorption or the metabolism of the study drug. Further drop‐out from PK analysis was based on missing samples hindering an unbiased estimation of (AUC_0‐24_) or the failure to achieve a stable Tac trough level (C_0_). The PK variables describing the cumulative dose per study period/period days (D_AV_), and conversion ratios (C_0_/dose_SS_, AUC_0‐24_/dose_SS_) were the primary endpoints of the study. PK variables assessing bioavailability in terms of extent and rate of exposure (area under the curve from 0 to 24 h [AUC_0–24_], time to peak concentration [t_max_], peak‐to‐trough fluctuation [PTF, which is defined as the fluctuation within a dosing interval in the steady state and is calculated as the quotient of the difference between the maximum and minimum plasma concentration and the mean plasma level], highest concentration determined in the measuring interval/the final dose when the predefined C_0_ at steady state was achieved [C_max_/dose_SS_]) served as secondary endpoints.

### 2.6 Safety analysis

All patients receiving at least once the study medication were included in the safety review. Safety was evaluated by investigating the incidence of reported adverse events (AEs) at regular intervals throughout the clinical trial and by assessing changes in physical examination findings, vital signs, and blood test parameters.

### 2.7 Statistical analysis

All statistical calculations were carried out using SAS language and procedures (SAS 8.2 or higher version SAS‐Institute Inc.). The arithmetic mean (mean), the standard deviation (SD), coefficient of variation (CV), minimum (min), maximum (max), and median (med) were reported for each variable. Additionally, the geometric mean (GeoM) was determined for concentration‐related parameters and, in accordance with the multiplicative model, the coefficient of variation of the GeoM was calculated as CV = (exp(*δ*²) ‐ 1)1/2, with *δ*² = variance of log‐transformed data. The individual patients' differences were preferably reported for the t_max_ while the patient's ratios are given for all other parameters. Analysis of variance (ANOVA) was performed for the following endpoints: D_AV_, AUC_0‐24_/dose_ss_, C_0_/dose_ss_, AUC_0‐24_, C_max,_ and C_0_. The sample size estimation was based on the endpoint of the AUC_0‐24_/dose, while data on the intra‐ or interpatient variability were not available for the primary endpoint of equivalent dose. A sample size of *n* = 20 patients was considered sufficient to determine the D_AV_.

## 3 RESULTS

### 3.1 Patient recruitment and study population

During the enrollment phase from August 31, 2016, to April 1, 2019, 189 patients underwent liver transplantation at the University Medical Center Hamburg‐Eppendorf. All pediatric liver transplant recipients (*n* = 61) and patients with a combined liver‐kidney transplant (*n* = 7) were excluded from participation in the study. The remaining liver transplant recipients were generally considered for the study; reasons for exclusion were (1) impaired kidney function requiring renal replacement therapy (2) poor graft function (INR > 2.0) or abnormal blood tests (thrombocytopenia < 20 10 × 9/L, leukopenia < 1.0 1 × 9/L) at the time of randomization, (3) the patient's inability to take oral medication, (4) the patient's refusal to participate in this very complex study, (5) long‐distance/long journey time between the patient's home and the transplant center, making the frequent follow‐up study visits impossible, and (6) unavailability of the liver transplant recipient to participate in the 24‐h PK profiles based on the medical need of an inpatient rehabilitation program, which is usually located far away from our transplant center.

**Figure 2:**
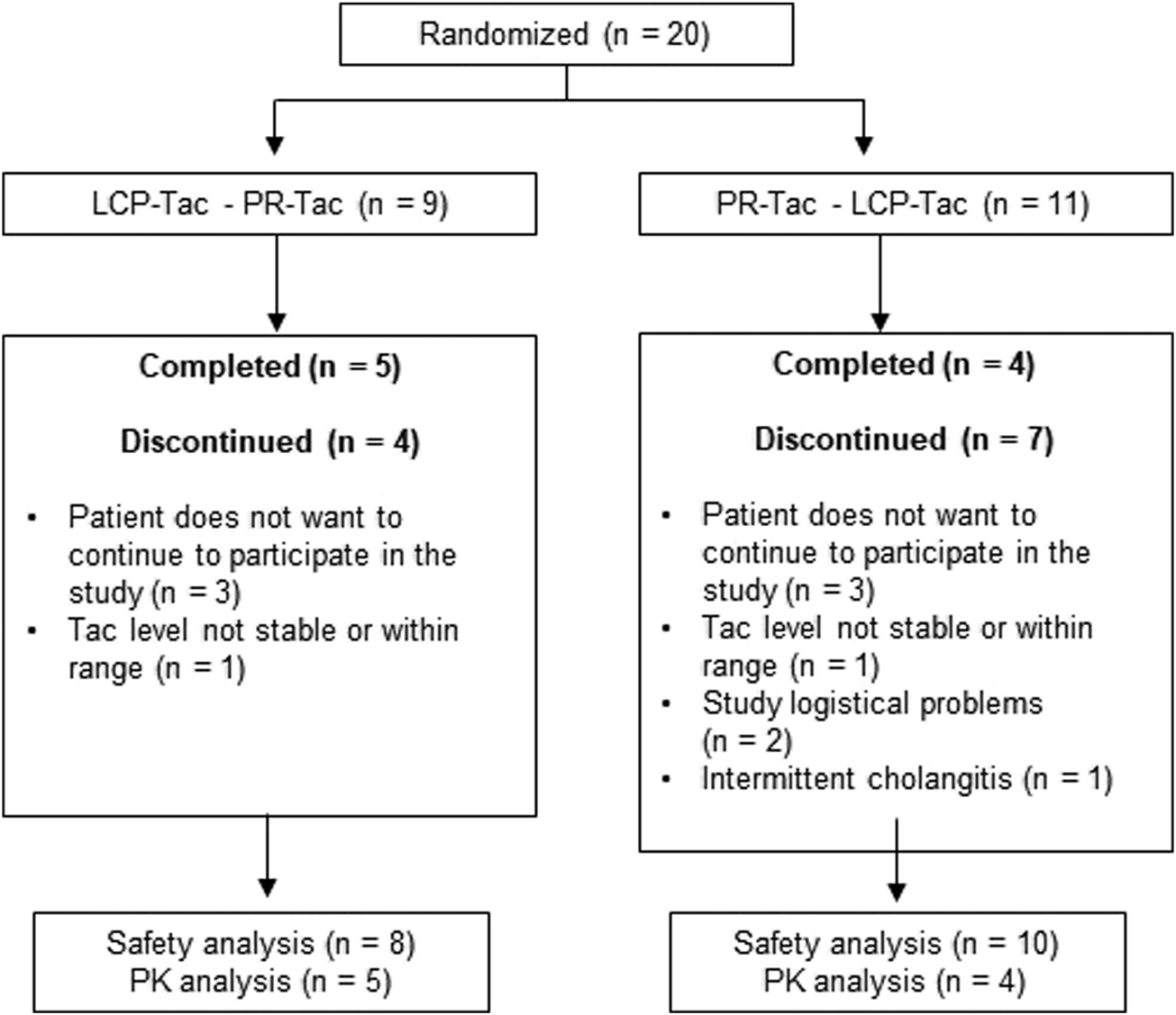
Patient enrollment

Hence, 20 patients were screened and included for participation in this study. Eighteen patients (90%) received the study medication at least once and were, therefore, included in the safety population (SAF). Nine patients (45%) completed the study, 11 patients were excluded from the PK population for various reasons (Figure 2).

### 3.2 Demographic and baseline characteristics

The majority of patients in both study groups were male with a mean (SD) age of 54.3 (10.34) years (Table 1). Ethnicity was White in 88.9% and Asian in the remaining 11.1%. The mean (SD) weight and body mass index (BMI) at baseline were 81.4 (12.53) kg and 26.7 (4.22), respectively. Four participants of the SAF cohort tested positive for hepatitis C (22.2%) and one liver transplant recipient was hepatitis B antigen positive (5.6%), while all donors were negative for both hepatitis C and hepatitis B antigen.

**Table 1:** Demographic and baseline characteristics of the safety population (SAF)

	PR‐Tac ‐ LCP‐Tac (*n* = 10)	LCP‐Tac ‐ PR‐Tac (*n* = 8)	Total (*n* = 18)
Sex, male, *n* (%)	8 (80.0%)	6 (75.0%)	14 (77.8%)
Age (years)			
Mean (SD)	50.6 (10.34)	58.9 (8.87)	54.3 (10.34)
Median	51.0	59.0	55.0
Minimum to maximum	31.0–63.0	46.0–71.0	31.0–71.0
Race, *n* (%)			
White	9 (90.0%)	7 (87.5%)	16 (88.9%)
Other	1 (10.0%)	1 (12.5%)	2 (11.1%)
Height (cm)			
Mean (SD)	176.1 (6.82)	173.3 (5.31)	174.8 (6.20)
Median	176.0	174.0	175.0
Minimum to maximum	163.0–186.0	165.0–180.0	163.0–186.0
Weight (kg)			
Mean (SD)	87.7 (12.0)	84.8 (13.16)	81.4 (12.53)
Median	77.2	88.5	80.6
Minimum to maximum	65.0–99.9	59.0–99.0	59.5–99.9
BMI (kg/m^2^)			
Mean (SD)	25.5 (4.20)	28.2 (3.97)	26.7 (4.22)
Median	24.3	28.3	26.2
Minimum to maximum	20.6–34.6	21.9–33.1	20.6–34.6
labMELD			
Mean (SD)	19.9 (7.8)	16.0 (9.16)	
Median	19.5	15.0	
Minimum to maximum	11–35	7–37	
*Transplant information*			
Donor age (years)			
Mean (SD)	52.0 (15.63)	62.5 (9.10)	56.7 (13.87)
Median	51.5	64.0	58.0
Minimum to maximum	27.0–77.0	50.0–75.0	27.0–77.0

Abbreviation: SD, standard deviation.

### 3.3 PK outcomes

**Figure 3:**
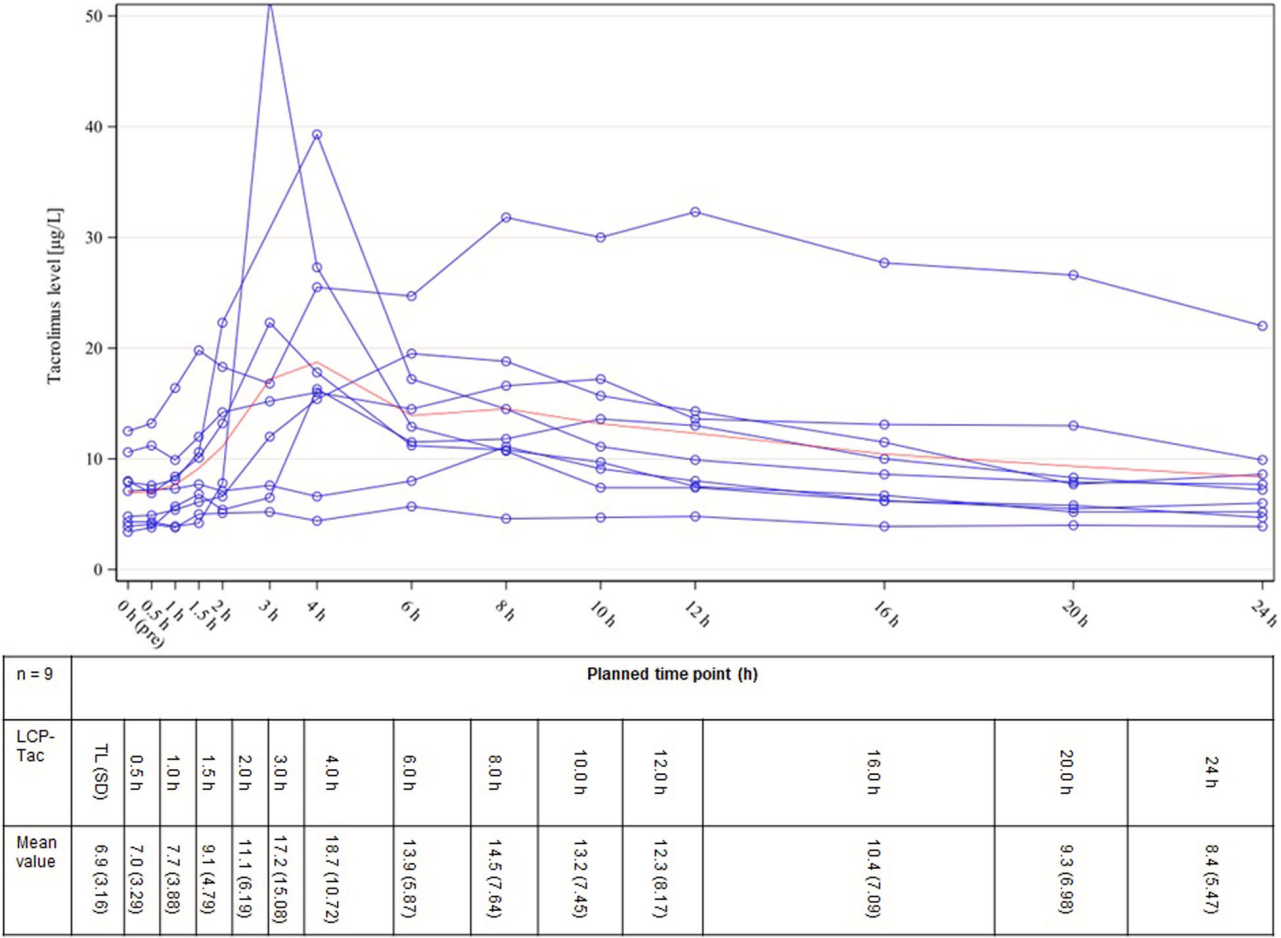
LCP‐Tac: individual plasma Tac concentrations (blue lines) and mean values (red curve). TL, trough level

**Figure 4:**
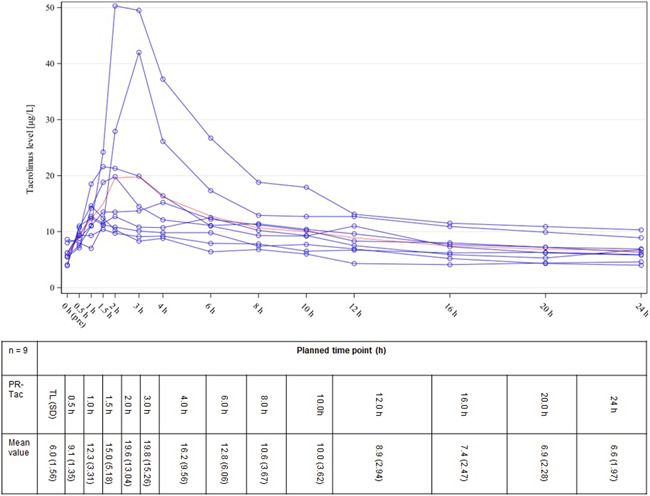
PR‐Tac: individual plasma Tac concentrations (blue lines) and mean values (red curve). TL, trough level

Nine patients completed both 14‐day study intervals with LCP‐Tac and PR‐Tac including plasma profiles, resulting in a total of 18 Tac PK profiles. Figures 3 and 4 illustrate the plasma profiles for the individual patients as well as the mean concentration of LCP‐Tac or PR‐Tac, respectively. The results of the absolute and relative bioavailability with the extent and rate of exposure calculated as C_0(LCP‐Tac)_/C_0(PR‐Tac)_, AUC_0‐24(LCP‐Tac)_/AUC_0‐24(PR‐Tac),_ and C_max(LCP‐Tac)_/C_max(PR‐Tac)_ are presented in Table 2. Adjusted GeoM ratios ranging between 100% and 115% indicated that the mean systemic exposure to LCP‐Tac was similar to that of PR‐Tac with no significant difference between the mean concentrations. However, the time point of maximum blood concentration (t_max_) was reached significantly earlier for PR‐Tac (median of 1.5 h) versus LCP‐Tac (median of 6.0 h) (location shift [90% CI]: 4.0 [2.0, 6.5]). Moreover, the PTF tended to be higher for PR‐Tac versus LCP‐Tac with adjusted GeoMs of 1.1 and 1.4, respectively (adjusted GeoM ratio: 84.3% [90% CI: 46.6, 152.5]). Taken together, the profile of the mean concentrations indicates a flattened kinetic of LCP‐Tac compared to PR‐Tac, especially in the first 3 h after drug intake (Figures 3 and 4).

**Table 2:** LCP‐Tac versus PR‐Tac: ANOVA with log‐transformed data

	Adjusted GeoM		Adjusted GeoM ratio (90% CI)	ANOVA‐CV (%)
PK variable	LCP‐Tac	PR‐Tac		
Pre‐dose Tac trough level at steady state (C_0_)	6.6	5.8	113.7 (92.2, 140.1)	23.6
Area under the curve in the last dosing interval at steady state (AUC_0–24 h_)	262.7	230.7	113.9 (84.1, 154.3)	34.8
Peak blood concentration of Tac (C_max_)	20.2	19.2	104.8 (63.2, 174.0)	61.2
Peak trough fluctuation (PTF)	1.1	1.4	84.3 (46.6, 152.5)	73.9
C_0_/daily dose at steady state (C_0_/dose_ss_)	0.9	0.5	176.2 (131.6, 235.8)	33.3
AUC_0–24 h_/dose_ss_	37.6	21.3	176.5 (139.0, 224.1)	27.0

Abbreviations: ANOVA, analysis of variance, ANOVA‐CV, intra‐individual variation; GeoM, geometric mean.

Regarding the required medication dose, the ratio C_0_/dose_SS_ was significantly higher for LCP‐Tac (0.9 vs. 0.5 for LCP‐Tac vs. PR‐Tac, adjusted GeoM ratio 176.2% [131.6%–235.8%]), which implies that the administered dose of LCP‐Tac was lower in relation to the predose concentration at steady state (C_0_). The same applied to the ratio AUC_0‐24_/dose_ss_ 37.6 versus 21.3 LCP‐Tac versus PR‐Tc, adjusted GeoM ratio 176.5% (139.0%–224.1%), which confirms a lower need for LCP‐Tac to achieve the same AUC_0‐24_. The average cumulative dose per day (D_AV_)—one of the primary endpoints of the study—was lower for LCP‐Tac (8.7 mg) than for PR‐Tac (11.7 mg). For D_AV_, the untransformed values were used and, therefore, the adjusted mean difference was calculated with ‐2.9 (90% CI: −5.9, 0.1).

### 3.4 Safety

Of the 18 patients included in the safety cohort, 15 had received at least one LCP‐Tac and 13 patients had received at least one dose of PR‐Tac. Overall, 16 patients (88.9%) experienced AEs and a total of 62 AEs were documented. Twenty‐six AEs were reported in eight patients (8/15 = 53.3%) during treatment with LCP‐Tac and 36 AEs occurred in 12 patients treated with PR‐Tac (12/13 = 92.3%). The most common AEs under both treatment regimens were infections/microbial colonization (38.9%), hepatobiliary complications, in particular cholangitis/cholestasis (27.8%), and central nervous system (CNS) disturbance such as dizziness, tremor, and headache (27.8%). Overall, most AEs were categorized as mild to moderate. Five patients experienced serious adverse events (SAE, *n* = 10) during the trial period. All SAEs were unrelated to the study drugs, and all 10 SAEs resolved. None of the study participants lost their graft or died during the study.

## 4 DISCUSSION

The present study is a randomized cross‐over PK study comparing the innovative once‐daily Tac formulation LCP‐Tac with the established once‐daily Tac formulation PR‐Tac in adult de novo liver transplant recipients. Comparing the ratio between C_0_ or the AUC_0‐24h_ to the daily dose, which served as a measure of relative oral bioavailability, LCP‐Tac demonstrated a better bioavailability than PR‐Tac. Additionally, treatment with LCP‐Tac resulted in a longer t_max_ and fewer PTF, which contributes to a potentially more stable and flattened PK profile of LCP‐Tac compared to PR‐Tac. Similar characteristics of LCP‐Tac, as opposed to IR‐Tac in liver transplant recipients, have been reported by Alloway et al.^12^ Moreover, Kamar et al.^13^ recently demonstrated in a PK study in de novo kidney transplant recipients that LCP‐Tac was associated with significantly less PTF, a longer t_max_, lower *C*_max_, and higher AUC than PR‐Tac.^13^ Hence, the different PK properties of LCP‐Tac imply a reduced risk for over ‐or under‐dosing of Tac and may also help to diminish the likelihood of Tac‐associated toxicities. Indeed, various studies have shown a correlation between CNI exposure and the incidence of complications after transplantation, including impaired kidney function, infections, or de novo malignancy.^14^ Recently, an association between high tacrolimus peak levels and the occurrence of PTLD in pediatric LT recipients has been published.^15^ Hence, the fewer PTF and the lower peak level of LCP‐TAC may imply a lower incidence of infections and de novo malignancies, especially PTLD.

One of the key messages of our study is the greater bioavailability of LCP‐Tac compared to PR‐Tac; consequently, lower doses of LCP‐Tac are required to achieve equivalent trough levels as opposed to PR‐Tac. A dose reduction of 20%–30% resulting in a conversion ratio of about 0.7 is well known from comparative pharmacological studies between LCP‐Tac and IR‐Tac in both kidney and liver transplantation,^12^, ^16^, ^17^ but data are scarce for the direct comparison of the once‐daily tacrolimus preparations LCP‐TAC versus PR‐TAC. A previous conversion study described a significant decrease of the mean daily Tac dose after switching from PR‐Tac to LCP‐Tac in stable liver transplant recipients, while the mean Tac trough level initially increased before approaching the preconversion level after 6 months. Baccarani et al. retrospectively compared LCP‐Tac with PR‐Tac in *de novo* liver transplant recipients focusing on administered daily dose and therapeutic trough levels during the first 30 days after transplant. Therapeutic trough levels were obtained faster under treatment with LCP‐Tac and, additionally, patients given LCP‐Tac required a 25% lower median dose to maintain the same therapeutic trough levels as patients treated with PR‐Tac.^18^ With a significantly higher ratio C_0_/dose_ss_ for LCP‐Tac compared to PR‐Tac in our work presented here, we confirm that less dose of LCP‐Tac is required to achieve a comparable trough level. Furthermore, a lower cumulative Tac dose per day was observed in our de novo liver transplant recipients receiving LCP‐Tac than in patients administered PR‐Tac.

LCP‐Tac and PR‐Tac exhibited a similar safety profile, with no new safety concerns observed. Overall, fewer AEs/SAEs were reported during administration of LCP‐Tac than under treatment with PR‐Tac. However, based on the cross‐over design of the study, it was not possible to clearly assign the AEs and SAEs to one of the Tac formulations. Yet, both treatments were well tolerated considering that only one event (mild in severity) was classified as drug‐related and none of the 10 serious AEs were categorized as drug‐related. Finally, a short‐term PK study does not aim to investigate side effects, and high rates of AEs and SAEs are well known from several drug studies after liver transplantation.^19^, ^20^

Unfortunately, we experienced a high drop‐out rate based on the study design with the inclusion of patients in the very vulnerable period early after LT. Only half of the enrolled patients (*n* = 9) successfully completed this PK study. However, the statistical analysis was adjusted accordingly. Furthermore, the cross‐over design not only minimized the risk of bias on bioabsorption by intraindividual differences, but also doubled the number of PK analyzes within the same small number of patients allowing meaningful statistical data analysis. Still, the small study population remains a major limitation of our study and the results of our observational, monocentric study should be interpreted with caution.

Taken together, minimizing side effects, the number of immunosuppressive agents and the dose of each of these immunosuppressive drugs are among the most important goals of successful immunosuppression in the liver transplant recipient, ultimately to prolong both graft and patient survival. Innovative formulations of Tac, which offer a better bioavailability or PK profile than conventional prolonged‐release formulations, can, therefore, aid to maintain therapeutic Tac levels with the potential benefit of reducing drug‐related toxicity or risk of organ rejection. Despite the limitations of the present work, available data of a more consistent and flattened PK profile and especially better bioavailability of LCP‐Tac are confirmed in our population of de novo LT patients.

## ETHICS STATEMENT

The appropriate institutional review board (IRB) approval is stated. The study was approved by the Ethics Committee of the University Medical Center Hamburg‐Eppendorf, Germany. All patients gave written consent for participation in the study.

## AUTHOR CONTRIBUTIONS

Uta Herden had the idea and designed the study and wrote the paper. Martina Sterneck, Bettina M. Buchholz, and Eike G. Achilles wrote the manuscript and performed a critical review of the manuscript. Armin Ott took responsibility for the integrity of the data and the accuracy of the data analysis. Lutz Fischer was involved in the study design. All authors contributed to data acquisition or data interpretation, reviewed, and approved the final version.

## Data Availability

The data that support the findings of this study are available from the corresponding author upon reasonable request.
